# Metabolic and Microbiota Measures as Peripheral Biomarkers in Major Depressive Disorder

**DOI:** 10.3389/fpsyt.2018.00513

**Published:** 2018-10-22

**Authors:** Rachael Horne, Jane A. Foster

**Affiliations:** ^1^Department of Psychiatry & Behavioural Neurosciences, McMaster University, Hamilton, ON, Canada; ^2^Department of Psychiatry, St. Michael's Hospital, Toronto, ON, Canada

**Keywords:** gut-brain axis, microbiome, leptin, ghrelin, major depression (MDD)

## Abstract

Advances in understanding the role of the microbiome in physical and mental health are at the forefront of medical research and hold potential to have a direct impact on precision medicine approaches. In the past 7 years, we have studied the role of microbiota-brain communication on behavior in mouse models using germ-free mice, mice exposed to antibiotics, and healthy specific pathogen free mice. Through our work and that of others, we have seen an amazing increase in our knowledge of how bacteria signal to the brain and the implications this has for psychiatry. Gut microbiota composition and function are influenced both by genetics, age, sex, diet, life experiences, and many other factors of psychiatric and bodily disorders and thus may act as potential biomarkers of the gut-brain axis that could be used in psychiatry and co-morbid conditions. There is a particular need in major depressive disorder and other mental illness to identify biomarkers that can stratify patients into more homogeneous groups to provide better treatment and for development of new therapeutic approaches. Peripheral outcome measures of host-microbe bidirectional communication have significant translational value as biomarkers. Enabling stratification of clinical populations, based on individual biological differences, to predict treatment response to pharmacological and non-pharmacological interventions. Here we consider the links between co-morbid metabolic syndrome and depression, focusing on biomarkers including leptin and ghrelin in combination with assessing gut microbiota composition, as a potential tool to help identify individual differences in depressed population.

Major depressive disorder (MDD) is a debilitating disorder that affects nearly 15% of the general population and accounts for the greatest disability burden of any disease (1). One of the major challenges in treating MDD is the lack of understanding of the underlying etiology of the disorder. The clinical heterogeneity observed in MDD makes it difficult to select the best treatment approach for an individual (2). Additionally, upwards of 60% of MDD patients will experience at least some form of treatment resistance over the course of the disease (3), with only one third of MDD suffers achieving remission even with optimal pharmacological and patient treatment (4). The onset and progression of depression is thought to result from a complex combination of genetic, environmental and neurochemical factors that differ considerably across patient populations such that the search for robust biomarkers to both characterize depression subtypes and predict treatment response is at the forefront of clinical psychiatry (5, 6).

Recent work over the last two decades has revealed the bidirectional communication between the central nervous system, enteric nervous system and the gastrointestinal tract, often referred to as the gut brain axis (7–12). Emerging evidence now supports the role of the gut microbiota in influencing behavior with specific links to MDD. The diverse population of the gut microbiota has been shown to be heterogeneous between individuals (13, 14), with dominate factors, such as diet (15), environment and host genetics (16–18) shaping the overall composition and function. Heterogeneity of depression has limited the success of efforts aimed at identifying clinical markers of treatment response and clinical subtypes. Treatment and diagnosis of MDD is also complicated by increasingly common comorbidities, resulting in another layer of heterogeneity in the MDD population. Therefore, the identification of robust biomarkers to both identify individual differences within the MDD population, as well as stratify patients into more homogenous subgroups is of critical importance. This approach is utilized by the Canadian Biomarker Integration Network in Depression (www.canbind.ca) that aims to shorten the time that it takes to match patients with the best treatment. By combining multiple molecular markers that can be measured in blood with clinical, imaging, or EEG researchers can identify and link biomarkers to clinical presentation and help predict treatment response (5).

Metabolic syndrome (MetS) is a well-documented in MDD patients, with the risk of MetS in depressed patient at 1.5 times higher than in the non-depressed population (19). Moreover, the prevalence of MetS is 58% higher in psychiatric patients than in the general population (20), with prominent anorexigenic and orexigenic hormones leptin and ghrelin identified to be associated with psychiatric disorders including schizophrenia, bipolar disorder and major depression (21–23). A role for the microbiome in metabolic syndrome has received attention, with studies demonstrating a role for gut microbiota in features of MetS, such as obesity, diabetes, dyslipidemia and hypertension (24, 25). Here we consider the literature related to leptin and ghrelin in MDD and how these molecular markers in combination with gut microbiota have potential to identify individual differences in patients and provide measures of the gut brain axis in MDD.

## Microbiota-brain interactions are important in depression

Research focused on the gut brain axis has gained momentum in recent years and garnered attention from both the scientific community, the public, and the media, with particular interest in understanding how microbes may influence mood. Accumulating evidence from preclinical work in rodents supports a connection between stress, microbiota, and stress-related behaviors (26–36), however, only a handful of studies have examined gut bacteria in individuals with major depressive disorder (7–9, 37–39). Using 16S rRNA gene sequencing, the composition of fecal microbiota in depressed patients was shown to be different from control samples (Table 1). The specific taxa differences observed in these studies varied, in part related to differences in sample size and analytical methods, but also related to the heterogeneity in the clinical populations recruited including age, BMI, smoking status, medication, clinical features, and severity of disease (8, 9, 37, 38). Inter-individual differences in microbiota composition in healthy individuals is ~90% (13, 14). Understanding how individual differences in microbiota influences individual differences in health and disease including MDD and other psychiatric disorders is needed.

**Table 1:** Bacterial taxa differences at the family and genus level observed in individuals with major depressive disorder.

**References**	**Family**	**Genus**
**Differences in relative abundance**		
Naseribafrouei et al. (38)	Lacnopiraceae (down)	*Alistipes* (up)
		*Oscillibacter* (up)
Jiang et al. (37)	Acidaminoccocaceae (up)	*Alistipes* (up)
Mothur metastats	Enterobacteriaceae (up)	*Blautia* (up)
	Fusobacteriaceae (up)	*Clostridibum XIX* (up)
	Porphyromonadaceae (up)	*Lachnospiacea* (up)
	Rikenellaceae (up)	*Megamonas* (up)
	Bacteroidaceae (down)	*Parabacteroides* (up)
	Erysipelotrichaceae (down)	*Parasutterella* (up)
	Lacnopiraceae (down)	*Phascolarctobacterium* (up)
	Prevotellaceae (down)	*Oscillibacter* (up)
	Ruminococcaceae (down)	*Roseburia* (up)
	Veillonellaceae (down)	*Bacteroides* (down)
		*Dialister* (down)
		*Faecalibacterium* (down)
		*Prevotella* (down)
		*Ruminococcus* (down)
Jiang et al. (37)	*Polphyromonadaceae* (up)	*Alistipes* (up)
LefSe LDA	*Eneterobacteriaceae* (up)	*Parabacteroides* (up)
Alpha leve = 0.05	*Rikenellaceae* (up)	*Butyricimonas* (up)
Effect size threshold = 2	*Erysipelotrichaceae* (up)	*Flavonifractor* (up)
	*Peptostreptococcaceae* (down)	*Haemophilus* (down)
	*Pasterueliaceae* (down)	*Dialister* (down)
	*Ruminococcaceae* (down)	*Faecalibacterium* (down)
		*Escherichia shigella* (down)
		*Ruminococcus* (down)
Kelly et al. (8)	Prevoellaceae (down)	*Prevotella* (down)
Mann-Whitney U test	Thermoanaerobacteriaceae (up)	*Dialister* (down)
FDR adjusted 10%		*Eggerthella* (up)
		*Holdemania* (up)
		*Gelria* (up)
		*Turicibacter* (up)
		*Paraprevotella* (up)
		*Anaerofilum* (up)
Lin et al. (9)
Wilcoxon's sign rank test		*Prevotella*
		*Steptococcus*
		*Clostridibum XIX*
Zheng et al. (39)	Actinomycineae (up)	*Parvimonas* (up)
Random forest classifier	Coriobacterineae (up)	*Anerostipes* (up)
	Lactobacillaceae (up)	*Blautia* (up)
	Streptococcaceae (up)	*Dorea* (up)
	Clostridales incertae sedis XI (up)	*Lachnospiraceae incertae sedis* (up)
	Eubacteriaceae (up)	*Clostridium IV* (up)
	Lachnospiraceae (up)	*Alistipes* (down)
	Ruminococcaceae (up)	*Coproccus* (down)
	Erysipelotrichaceae in certae sedis (up)	*Clostridium XIVa* (down)
	Bacteroidaceae (down)	*Phascolarctobacterium* (down)
	Rikenellaceae (down)	*Megamonas* (down)
	Lachnospiraceae (down)	*Lachnospiraceae incertae sedis* (down)
	Acidaminococcaceae (down)	*Roseburia* (down)
	Vellonellaceae (down)	*Faecalibacterium* (down)
	Sutterellaceae (down)	

The compositional data gained from utilizing 16S rRNA gene sequencing in the MDD population represents an accessible biomarker, that provides both compositional data and, with recent advances in bioinformatics, can contribute functional information (40). The potential therapeutic benefits of treatments that target the microbiome, including probiotic and prebiotic administration, have begun to gain creditability for the treatment of psychiatric disorders (41) and the term psychobiotics is commonly used to classify products, such as probiotics and prebiotics, that when given in adequate amounts produce positive psychological effects (42, 43). Several studies show a benefit of probiotic consumption in healthy individuals including improved mood (44), a beneficial effect on anxiety and depressive measures as well as reduced stress hormone levels (45). Less work has been conducted in MDD clinical populations (46), and work to date has not utilized gut-brain biomarkers to identify subgroups within MDD. Metabolism and metabolic markers are of interest in the microbiome field. Here we selected two well-known metabolic markers that have been well-studied in MDD populations and consider their potential as biomarkers in MDD.

## Leptin as a potential biomarker of gut-brain interactions in MDD

Leptin is an adipocyte derived hormone, with a known role in regulating fat mass storage and energy homeostasis. Leptin circulates as a 16 kDa protein, where it crosses the blood brain barrier (BBB) and interacts with multiple regions of the brain including the hypothalamus and hippocampus (47, 48). Over the last decade there has been increasing evidence of leptin's role in regulating mood (23). Work in animal models has revealed a complex role of circulating leptin along with leptin's receptor (lepR) expression levels throughout the brain. Deletion of the lepR in the hippocampus of rats results in depressive behavior (49), as well as inhibits the behavioral effect of serotonin reuptake inhibitor fluoxetine (50). Animal models of chronic stress have been found to reduce circulating leptin, as well as reliably produce depressive behavior. Systematic injection of leptin has been found to have a dose dependent reduction in depressive behaviors in chronically stress mice (51, 52). A study aimed at exploring the relationship between obesity and depression found that, a combination of diet induced obesity and chronic unpredictable mild stress (CUMS) resulted in increased leptin levels but a decrease in LepR expression, along with depressive behaviors (53). The disparity in leptin serum level and receptor expression may be more representative of what occurs in an obese human and may be contributing to the comorbidity of obesity and MDD.

Human studies examining leptin levels in MDD have been mixed, some finding elevated leptin in MDD (54, 55) while others finding decreased levels (56, 57). This may be due to the varying role of leptin in lean verse obese conditions but also represents how we expect a biomarker of individual differences to present in a clinical population. Under lean conditions leptin acts as an anti-obesity hormone, signaling through activation of leptin receptors at the hypothalamus to reduce feeding behavior (58). Obesity is characterized by an increase in circulating leptin and a decrease in leptin receptor expression, leading to leptin resistance and disrupted leptin signaling (59). Concurrently, alterations to appetite, as well as weight changes are known clinical features of MDD (2). Interestingly, two meta-analyses have found a significant association of elevated leptin with depression only when controlling for BMI (60, 61). There is also evidence to support the association between atypical features of depression, such as increase appetite and hyposomnia with elevated leptin levels (62, 63). A recent study aimed at identifying subgroups of depression, found that grouping un-medicated patients by increases or decreases in appetite revealed dramatic differences in metabolic signaling, immune signaling and functional brain activity differences (64). Leptin levels were significantly increased in MDD patients with increased appetite, compared to healthy controls or when compared to MDD individuals with decreased appetite, an observation that was not related to BMI (64). The increased appetite subgroup also had alteration to proinflammatory markers and decreases in orexigenic gut hormone ghrelin, suggesting that biological difference may contribute to differences in disease symptomology. Based on the work to date, leptin may be a useful biomarker in a particular subset of MDD patients and may aid in identifying individual differences.

Leptin levels are related to gut microbiota. The secretion of leptin by adipocytes is regulated by microbial-derived metabolites, specifically short-chain fatty acids (SCFA) that signal through GRP41/42 receptors (65). Studies have shown that the gut microbiota can influence leptin levels independent of food intake (66), and that prebiotic treatment can improve leptin sensitivity (67). Antibiotic use, which has been found as a risk factor for the development of depression has also been shown to reduce leptin levels in rodents (68). In line with the predicted relationship between bacterial populations and leptin levels, several studies have found that certain bacterial taxa correlate with circulating leptin levels. A study by Queipo-Ortuño et al. found that bacteria genera *Lactobacillus* and *Bifidobacterium* positively correlate with serum leptin levels, whereas *Clostridium, Bacteroides*, and *Prevotella* were negatively correlated. In a different study, obese and overweight pregnant women were found to have leptin levels that positively correlated with the abundance of the families Lachnospiraceae and Ruminococcaceae, highlighting the association between leptin levels and energy homeostasis (69). The impact of probiotic treatment on leptin levels within the context of depression has recently been evaluated (62). In mice, administration of *Pseudocatenulatum* reduced depressive behavior and improved leptin serum levels and receptor expression in the hippocampus and intestine of mice (59). Considering circulating leptin as a link to both MDD, metabolic disorders and the gut microbiota may advance its use as a biomarker to identify individual differences in MDD patients.

## Ghrelin as a potential biomarker of gut-brain interactions in MDD

Ghrelin is a gut peptide hormone that is produced by cholinergic cells in the gastrointestinal tract found predominately in the stomach (70). Acylated ghrelin circulates throughout the body and crosses the BBB, where it interacts with acylated ghrelin receptors (GHSR1), expressed by the hypothalamus (71). GHSR1 is also expressed in the dentate gyrus of the hippocampus, CA2 and CA3 regions, substratum nigria and ventral tegmental area (72). Due to the wide spread expression of GHSR1, ghrelin has been shown to play a role, in energy homeostasis, eating behavior, sleeping behavior (73), cognition, reward mechanisms (74), and mood (75), all of which can be altered in MDD. As with leptin, a body of work now supports the role of ghrelin in regulating mood, with close links to depression (73, 76). Ghrelin has a role in response to acute stress in both animals and humans, with acute stress resulting in elevated ghrelin levels and activation of the hypothalamus-pituitary axis (HPA) (77, 78). Preclinical work has shown that ghrelin inhibited the release of serotonin (79), as well as increased serotonin turnover (80), providing evidence of it potential role in serotonin imbalance observed in MDD. Animal studies have found behavioral effects following cerebral injection of ghrelin including decreased anxiety and depressive behaviors (81). It is suggested that ghrelin acts as a survival mechanism when animals are exposed to acute stress, to induce feeding behavior. In support of this suggestion, a study in 2012 showed the elevated ghrelin after acute stress attenuated anxiety-like behaviors, whereas prolonged stress led to chronic increased ghrelin levels, dysregulation of HPA axis and serotonin signaling as well as increased depressive behaviors (78).

Attempts to associate ghrelin levels in humans with MDD has also shown mixed results, with older studies indicating a decrease in ghrelin levels (82) while newer studies are finding an elevation of ghrelin associated with MDD (83–85). Ghrelin has been predicted to alter a number of genes involved in depression with a ghrelin polymorphism found to be associated with the development of depression (86). Three previous studies have shown that ghrelin may act as a measure of treatment response, finding elevated ghrelin levels in MDD non-responders, and a decrease of serum ghrelin levels associated with response to treatment (85, 87, 88). Serum ghrelin has been recently shown to act as a persistent biomarker for chronic stress exposure in both rodents and humans (89) with exposure to chronic stressors resulting in elevated acyl-ghrelin levels for at least 130 days in rats and 4.5 years in adolescent humans. Indicating that those with elevated ghrelin and MDD, may have a chronic stressor as an underlying mechanism of disease progression. Exposure to both chronic and acute stress results in elevated circulating cortisol levels. The same study identified subgroups of MDD based on appetite and found elevated ghrelin and cortisol levels in MDD patients with decreased appetite (64). This finding indicates that in the depressed population elevated ghrelin may have roles outside of increasing eating behavior and may interact to influence the HPA axis resulting in elevated stress response. Furthermore, work by Algul et al. found both acylated and deacylated serum ghrelin level were elevated in MDD patients and increases in ghrelin concentration significantly correlated with disease severity (84).

Ghrelin is primarily produced in the gut, with previous work establishing the role of the vagus nerve in mediating the communication of the peptide to the brain (90). Due to the proximity of the gut microbiota to ghrelin's central location, work has begun to explore the relationship between the gut microbiota and ghrelin expression. Notably, germ-free mice have lower levels of circulating ghrelin, with levels increasing beyond conventional mice after a period of fasting (91). Additionally, serum ghrelin levels significantly negatively correlate to genera *Bifidobacterium, Lactobacillus*, and *B. coccoides-Eubacterium rectale* and positively correlate with *Bacteroides* and *Prevotella* in rodents (92). Treatment with prebiotics has been found to alter ghrelin levels, with lean and obese mice exhibiting a positive response in ghrelin following prebiotic treatment (93). Metabolism of prebiotics by gut bacteria leads to increased SCFAs and, ghrelin production has been found to be regulated by SCFA signaling (94, 95). Activation of the fatty acid receptor 3 by butyrate reduced serum ghrelin levels (94) however; a recent study identified the role of bacteria-derived acetate in activating the parasympathetic nervous system and increasing ghrelin secretion (95). Further, gastric infusion of acetate dramatically increases ghrelin concentrations in plasma, but the effects were lost in vagotomised mice (95), indicating potential bidirectional communication from the gut to the brain in the control of ghrelin secretion from cholinergic cells. Due to ghrelin's increasingly recognize role in mediate mood and potential biomarker status for MDD, along with its connection to the gut microbiota, it makes an optimal biomarker to identify treatment response to prebiotic and probiotic treatment in the MDD population.

## Implication and future directions

While much excitement has been recently focused on the role of the gut microbiota in psychiatric disorders, there is a need to gain a better understand clinical heterogeneity in depressed individuals. Research on gut the brain axis has been rapidly progressing, by examining the relationship between the gut microbiota and metabolic states in healthy and depressed individuals, a better understanding of how microbes influence mood will be determine. As MDD commonly occurs with co-morbidities, it is important to evaluate how related factors contribute to disease development or progression. As the relationship between metabolic syndrome and depression is bilateral and suggested that the development of one often leads to the other (96) exploring metabolic endocrine signaling in the context of depression and gut microbiota will enable researchers and clinicians to gain a broader understanding of the underlying biological factors that may be contributing to MDD.

As neuroscientists, psychologists, and psychiatrists are starting to appreciate the importance of gut microbiota to mental health, there is a great opportunity to identify biomarkers associated with the gut-brain axis and thereby provide a better understanding of the aspects that may be modifiable with proper intervention in individuals with mental illness. By measuring: leptin and ghrelin levels, both within context of sex and BMI, and in conjunction with gut microbiota composition and MDD symptomology, researchers will be able to stratify the clinical population in more homogenous subgroups. The ability to identify a subgroup of the clinical MDD population based on metabolic status and gut microbiota composition would aid clinical trials to predict treatment response and for development of therapies that target the microbiome.

## Author contributions

JF and RH developed the framework for the mini-review. RH wrote the manuscript, JF edited the manuscript. Both authors approved its submission.

### Conflict of interest statement

The authors declare that the research was conducted in the absence of any commercial or financial relationships that could be construed as a potential conflict of interest.
